# Sharp Smith’s bounds for the gamma function

**DOI:** 10.1186/s13660-018-1620-3

**Published:** 2018-01-31

**Authors:** Xi-Qiao Li, Zhi-Ming Liu, Zhen-Hang Yang, Shen-Zhou Zheng

**Affiliations:** 1 School of Mechanical, Electronic and Control Engineering, Beijing Jiaotong University, Beijing, China; 2 Department of Science and Technology, State Grid Zhejiang Electric Power Company Research Institute, Hangzhou, China; 3 College of Science, Beijing Jiaotong University, Beijing, China

**Keywords:** 33B15, 26A48, 26D15, 26A51, Gamma function, Approximation formula, Inequality

## Abstract

Among various approximation formulas for the gamma function, Smith showed that

$$ \Gamma \biggl( x+\frac{1}{2} \biggr) \thicksim S ( x ) =\sqrt{2 \pi } \biggl( \frac{x}{e} \biggr) ^{x} \biggl( 2x\tanh \frac{1}{2x} \biggr) ^{x/2}, \quad x\rightarrow \infty , $$

which is a little-known but accurate and simple one. In this note, we prove that the function $x\mapsto \ln \Gamma ( x+1/2 ) - \ln S ( x ) $ is strictly increasing and concave on $( 0,\infty ) $, which shows that Smith’s approximation is just an upper one.

## Introduction

The Stirling formula

1.1 $$ n!\thicksim \sqrt{2\pi n}n^{n}e^{-n} $$

has many important applications in statistical physics, probability theory and number theory. Due to its practical importance, it has attracted much interest of many mathematicians and has also motivated a large number of research papers concerning various generalizations and improvements; see for example, Burnside’s [1], Gosper [2], Batir [3], Mortici [4].

The gamma function $\Gamma ( x ) =\int_{0}^{\infty }t^{x-1}e ^{-t}\,dt $ for $x>0$ is closely related to the Stirling formula, since $\Gamma (n+1)=n!$ for all $n\in \mathbb{N}$. This inspired some authors to also pay attention to find various better approximations for the gamma function; see, for instance, Ramanujan [5, p. 339], Windschitl (see Nemes [6, Corollary 4.1]), Yang and Chu [7], Chen [8].

More results involving the approximation formulas for the factorial or gamma function can be found in [9–23] and the references cited therein.

In this note, we are interested in Smith’s approximation formula (see [24, equation (42)]):

1.2 $$ \Gamma \biggl( x+\frac{1}{2} \biggr) \thicksim \sqrt{2\pi } \biggl( \frac{x}{e} \biggr) ^{x} \biggl( 2x\tanh \frac{1}{2x} \biggr) ^{x/2}:=S ( x ), \quad \text{as }x\rightarrow \infty . $$

It is easy to check that

$$ \Gamma \biggl( x+\frac{1}{2} \biggr) =\sqrt{2\pi } \biggl( \frac{x}{e} \biggr) ^{x} \biggl( 2x\tanh \frac{1}{2x} \biggr) ^{x/2} \biggl( 1+O \biggl( \frac{1}{x^{5}} \biggr) \biggr) , $$

which shows that the rate of $S ( x ) $ converging to $\Gamma ( x+1/2 ) $ as $x\rightarrow \infty $ is like $x^{-5}$. According to the comment in [8, (3.5)–(3.10)], it is well known that Smith’s approximation is an accurate but simple one for gamma function.

The aim of this short note is to further prove the Smith approximation $S ( x ) $ is an upper one. Our main result is stated as follows.

### Theorem 1

*The function*

$$ f ( x ) =\ln \Gamma \biggl( x+\frac{1}{2} \biggr) -\ln \sqrt{2 \pi }-x\ln x+x-\frac{x}{2}\ln \biggl( 2x\tanh \frac{1}{2x} \biggr) $$

*is strictly increasing and concave from* $( 0,\infty ) $ *onto* $( -\ln \sqrt{2},0 ) $.

## Proof of Theorem 1

To prove Theorem 1 we need the following two lemmas.

### Lemma 1

*The inequality*

2.1 $$ \psi^{\prime } \biggl( x+\frac{1}{2} \biggr) < \frac{4}{3} \frac{15x^{2}+4}{x ( 20x^{2}+7 ) } $$

*holds for all* $x>0$.

### Proof

Let

2.2 $$ f_{1} ( x ) =\psi^{\prime } \biggl( x+\frac{1}{2} \biggr) - \frac{4}{3}\frac{15x^{2}+4}{x ( 20x^{2}+7 ) }. $$

Using the recurrence formula [25, pp. 258–260]:

$$ \psi^{ ( n ) }(x+1)-\psi^{ ( n ) }(x)=\frac{ ( -1 ) ^{n}n!}{x^{n+1}}, $$

we have

$$\begin{aligned} f_{1} ( x+1 ) -f_{1} ( x ) =&\psi^{\prime } \biggl( x+ \frac{3}{2} \biggr) -\frac{4}{3 ( x+1 ) }\frac{15x^{2}+30x+19}{20x ^{2}+40x+27} \\ &{}-\psi^{\prime } \biggl( x+\frac{1}{2} \biggr) +\frac{4}{3} \frac{15x ^{2}+4}{x ( 20x^{2}+7 ) } \\ &{}-\frac{1}{ ( x+1/2 ) ^{2}}-\frac{4}{3 ( x+1 ) }\frac{15x ^{2}+30x+19}{20x^{2}+40x+27}+\frac{4}{3} \frac{15x^{2}+4}{x ( 20x ^{2}+7 ) } \\ =&\frac{144}{x ( x+1 ) ( 2x+1 ) ^{2} ( 20x ^{2}+7 ) ( 20x^{2}+40x+27 ) }>0. \end{aligned}$$

It then follows that

$$ f_{1} ( x ) < f_{1} ( x+1 ) < \cdots < \lim _{n\rightarrow \infty }f_{1} ( x+n ) =0, $$

which proves the desired inequality (2.1). □

### Lemma 2

*The inequality*

2.3 $$ \frac{\sinh^{2}t}{\cosh t}>\frac{t^{2} ( 21t^{2}+60 ) }{31t ^{2}+60} $$

*holds for all* $t>0$.

### Proof

It is obvious that the inequality what we consider is equivalent to

$$ f_{2} ( t ) = \bigl( 31t^{2}+60 \bigr) ( \sinh t ) ^{2}-t^{2} \bigl( 21t^{2}+60 \bigr) \cosh t>0. $$

Simplifying and expanding it in power series lead us to

$$\begin{aligned} 2f_{2} ( t ) =&60\cosh 2t+31t^{2}\cosh 2t-120t^{2}\cosh t-42t ^{4}\cosh t-31t^{2}-60 \\ =&60\sum_{n=0}^{\infty }\frac{2^{2n}}{ ( 2n ) !}t^{2n}+31 \sum_{n=1}^{\infty }\frac{2^{2n-2}}{ ( 2n-2 ) !}t^{2n} \\ &{}-120\sum_{n=1}^{\infty }\frac{1}{ ( 2n-2 ) !}t^{2n}-42 \sum_{n=2}^{\infty }\frac{1}{ ( 2n-4 ) !}t^{2n}-31t^{2}-60 \\ :=&\sum_{n=2}^{\infty }\frac{a_{n}}{ ( 2n ) !}t^{2n}, \end{aligned}$$

where

$$ a_{n}= \bigl( 62n^{2}-31n+120 \bigr) 2^{2n-1}-24n ( 2n-1 ) \bigl( 14n^{2}-35n+31 \bigr) . $$

It is easy to check that $a_{2}=a_{3}=0$ and $a_{4}=49\,184>0$. It remains to prove $a_{n}>0$ for $n\geq 5$.

To this end, it suffices to prove $b_{n}=2^{2n-1}-6n ( 2n-1 ) >0$ for $n\geq 5$, because the inequality

$$ \bigl( 62n^{2}-31n+120 \bigr) >4 \bigl( 14n^{2}-35n+31 \bigr) $$

is clearly valid for $n\geq 5$. We easily obtain

$$ b_{n+1}-4b_{n}=6 \bigl( 6n^{2}-7n-1 \bigr) >0 $$

for $n\geq 5$, which in combination with $b_{5}=242>0$ yields $b_{n}>0$ for $n\geq 5$. This completes the proof. □

Now we are in a position to prove Theorem 1.

### Theorem 1

Differentiating and simplifying yields

$$\begin{aligned}& f^{\prime } ( x ) = \psi \biggl( x+\frac{1}{2} \biggr) - \ln x- \frac{1}{2}\ln \biggl( 2x\tanh \frac{1}{2x} \biggr) + \frac{1}{2x \sinh ( 1/x ) }-\frac{1}{2}, \\& f^{\prime \prime } ( x ) = \psi^{\prime } \biggl( x+ \frac{1}{2} \biggr) +\frac{1}{2x^{3}}\frac{\cosh ( 1/x ) }{ \sinh^{2} ( 1/x ) }-\frac{3}{2x}. \end{aligned}$$

As an application of inequalities (2.1) and (2.3) it gives

$$\begin{aligned}& \begin{aligned} f^{\prime \prime } ( x ) &< \frac{4}{3}\frac{15x^{2}+4}{x ( 20x^{2}+7 ) }+\frac{1}{2x^{3}} \frac{\cosh ( 1/x ) }{\sinh^{2} ( 1/x ) }-\frac{3}{2x} \\ &=\frac{1}{2x^{3}}\frac{\cosh ( 1/x ) }{\sinh^{2} ( 1/x ) }-\frac{1}{6}\frac{60x^{2}+31}{x ( 20x^{2}+7 ) } \\ & \mathop{=}^{x=1/t}\frac{t^{3}}{2} \biggl( \frac{\cosh t}{ \sinh^{2}t}- \frac{31t^{2}+60}{t^{2} ( 21t^{2}+60 ) } \biggr) < 0. \end{aligned} \end{aligned}$$

Then it is deduced that

$$ f^{\prime } ( x ) >\lim_{x\rightarrow \infty }f^{\prime } ( x ) =0, $$

which in turn implies that

$$ -\frac{1}{2}\ln 2=\lim_{x\rightarrow 0^{+}}f ( x ) < f ( x ) < \lim _{x\rightarrow \infty }f ( x ) =0. $$

This completes the proof. □

## Corollaries and remarks

Using the increasing property of $f ( x+1/2 ) $ given in Theorem 1 and noting that

$$ f \biggl( \frac{1}{2} \biggr) =\ln \frac{\sqrt{e}}{\sqrt{\pi } ( \tanh 1 ) ^{1/4}}\quad \text{and}\quad f \biggl( \frac{3}{2} \biggr) = \ln \biggl( \frac{2e\sqrt{e}3^{3/4}}{27\sqrt{\pi }\tanh^{3/4} ( 1/3 ) } \biggr) , $$

we have the corollaries.

### Corollary 1

*The double inequality*

$$ \alpha_{1}< \frac{e^{x+1/2}\Gamma ( x+1 ) }{\sqrt{2\pi } ( x+1/2 ) ^{x+1/2} [ ( 2x+1 ) \tanh ( 1/ ( 2x+1 ) ) ] ^{ ( 2x+1 ) /4}}< 1 $$

*holds for all* $x>0$ *with the best constants* 1 *and* $\alpha_{1}=\sqrt{e/ \pi }/ ( \tanh 1 ) ^{1/4}\approx 0.99573$.

### Corollary 2

*The double inequality*

$$ \alpha_{2}< \frac{n!}{\sqrt{2\pi } ( ( n+1/2 ) /e ) ^{n+1/2} [ ( 2n+1 ) \tanh ( 1/ ( 2n+1 ) ) ] ^{ ( 2n+1 ) /4}}< 1 $$

*holds for all* $n\in \mathbb{N}$ *with the best constants* 1 *and*

$$ \alpha_{2}=\frac{2e\sqrt{e}3^{3/4}}{27\sqrt{\pi }\tanh^{3/4} ( 1/3 ) }\approx 0.99994. $$

By the decreasing property of $f^{\prime } ( x+1/2 ) $ given in Theorem 1 and the facts that

$$\begin{aligned}& f^{\prime } \biggl( \frac{1}{2} \biggr) =\frac{1}{\sinh 2}- \frac{1}{2} \ln ( \tanh 1 ) +\ln 2-\frac{1}{2}-\gamma \approx 0.027823, \\& f^{\prime } \biggl( \frac{3}{2} \biggr) =\frac{1}{3\sinh ( 2/3 ) }- \frac{1}{2}\ln \biggl( 3\tanh \frac{1}{3} \biggr) -\ln \frac{3}{2}+\psi ( 1 ) +\frac{1}{2}\approx 0.00016946, \end{aligned}$$

the following corollaries are immediate.

### Corollary 3

*For* $x>0$, *the inequalities*

$$\begin{aligned}& \frac{1}{2}+\frac{1}{2}\ln \biggl( ( 2x+1 ) \tanh \frac{1}{2x+1} \biggr) -\frac{1}{ ( 2x+1 ) \sinh ( 2/ ( 2x+1 ) ) } \\& \quad < \psi ( x+1 ) -\ln \biggl( x+\frac{1}{2} \biggr) \\& \quad < \beta_{1}+\frac{1}{2}\ln \biggl( ( 2x+1 ) \tanh \frac{1}{2x+1} \biggr) -\frac{1}{ ( 2x+1 ) \sinh ( 2/ ( 2x+1 ) ) } \end{aligned}$$

*hold*, *where the constants* $1/2$ *and*

$$ \beta_{1}=\ln 2-\frac{1}{2}\ln ( \tanh 1 ) +\frac{1}{\sinh 2}- \gamma \approx 0.52782 $$

*are the best possible*.

### Corollary 4

*Let* $H_{n}=\sum_{k=1}^{n}$ *for* $n\in \mathbb{N}$. *The inequalities*

$$\begin{aligned}& \biggl( \frac{1}{2}+\gamma \biggr) +\frac{1}{2}\ln \biggl( ( 2n+1 ) \tanh \frac{1}{2n+1} \biggr) -\frac{1}{ ( 2n+1 ) \sinh ( 2/ ( 2x+1 ) ) } \\& \quad < H_{n}-\ln \biggl( n+\frac{1}{2} \biggr) \\& \quad < \beta_{2}+\frac{1}{2}\ln \biggl( ( 2n+1 ) \tanh \frac{1}{2n+1} \biggr) -\frac{1}{ ( 2n+1 ) \sinh ( 2/ ( 2n+1 ) ) } \end{aligned}$$

*hold*, *where* $1/2+\gamma \approx 1.0772$ *and*

$$ \beta_{2}=\frac{1}{3\sinh ( 2/3 ) }-\frac{1}{2}\ln \biggl( 3 \tanh \frac{1}{3} \biggr) -\ln \frac{3}{2}+1\approx 1.0774 $$

*are the best possible constants*.

Finally, as a by-product of Lemma 1, we draw the following conclusion.

### Theorem 2

*Let* *g* *be defined on* $( 0,\infty ) $ *by*

$$ g ( x ) =\ln \Gamma \biggl( x+\frac{1}{2} \biggr) - \biggl[ \frac{1}{2}\ln 2\pi +\frac{16}{21}x\ln x+\frac{5x}{42}\ln \biggl( x ^{2}+\frac{7}{20} \biggr) -x-\frac{\sqrt{35}}{42} \operatorname{arccot} \biggl( \sqrt{\frac{20}{7}}x \biggr) \biggr] . $$

*Then* *g* *is strictly increasing and concave on* $( 0,\infty ) $.

### Proof

Differentiation yields

$$\begin{aligned}& g^{\prime } ( x ) =\psi \biggl( x+\frac{1}{2} \biggr) - \biggl[ \frac{5}{42}\ln \biggl( x^{2}+\frac{7}{20} \biggr) + \frac{16}{21}\ln x \biggr] , \\& g^{\prime \prime } ( x ) =\psi^{\prime } \biggl( x+ \frac{1}{2} \biggr) -\frac{4}{3}\frac{15x^{2}+4}{x ( 20x^{2}+7 ) }=f_{1} ( x ) < 0, \end{aligned}$$

where the inequality holds due to Lemma 1. This completes the proof. □

### Remark 1

Theorem 2 gives a new approximation for the gamma function

$$ \Gamma \biggl( x+\frac{1}{2} \biggr) \thicksim \sqrt{2\pi }x^{26x/21} \biggl( x^{2}+\frac{7}{20} \biggr) ^{5x/42}\exp \biggl[ -x-\frac{ \sqrt{35}}{42}\operatorname{arccot} \biggl( \sqrt{\frac{20}{7}}x \biggr) \biggr] , $$

as $x\rightarrow \infty $, which satisfies

$$ \Gamma \biggl( x+\frac{1}{2} \biggr) =\sqrt{2\pi }x^{26x/21} \biggl( x ^{2}+\frac{7}{20} \biggr) ^{5x/42}\exp \biggl[ -x- \frac{\sqrt{35}}{42}\operatorname{arccot} \biggl( \sqrt{\frac{20}{7}}x \biggr) \biggr] \bigl( 1+O \bigl( x^{-5} \bigr) \bigr) . $$

### Remark 2

Theorem 2 also offers an asymptotic formula for the psi function

$$ \psi \biggl( x+\frac{1}{2} \biggr) \thicksim \frac{5}{42}\ln \biggl( x ^{2}+\frac{7}{20} \biggr) +\frac{16}{21}\ln x\quad \text{as }x \rightarrow \infty . $$

Furthermore, by replacing *x* with $x+1/2$, we have the following sharp inequalities:

3.1 $$\begin{aligned}& \frac{5}{42}\ln \biggl( x^{2}+x+\frac{3}{5} \biggr) + \frac{16}{21} \ln \biggl( x+\frac{1}{2} \biggr) \\& \quad < \psi ( x+1 ) < \lambda_{0}+\frac{5}{42}\ln \biggl( x^{2}+x+ \frac{3}{5} \biggr) +\frac{16}{21}\ln \biggl( x+ \frac{1}{2} \biggr) \end{aligned}$$

for $x>0$ with the best constant

$$\begin{aligned}& \lambda_{0}=\frac{16}{21}\ln 2-\frac{5}{42}\ln \frac{3}{5}-\gamma \approx 0.011709; \\& \gamma +\frac{5}{42}\ln \biggl( n^{2}+n+\frac{3}{5} \biggr) + \frac{16}{21}\ln \biggl( n+\frac{1}{2} \biggr) \\& \quad < H_{n}< \lambda_{0}+\gamma +\frac{5}{42}\ln \biggl( n^{2}+n+ \frac{3}{5} \biggr) +\frac{16}{21}\ln \biggl( n+ \frac{1}{2} \biggr) \end{aligned}$$

for $n\in \mathbb{N}$ with the best constant

$$ \lambda_{1}=1-\frac{16}{21}\ln \frac{3}{2}- \frac{5}{42}\ln \frac{13}{5}-\gamma \approx 0.00010718. $$

Inequalities (3.1) first appeared in [26, Corollary 3.4].

## Conclusions

In this note, we mainly presented an upper bound of Smith’s approximation in accordance with the fact that the function $x\mapsto \ln \Gamma ( x+1/2 ) - \ln S ( x ) $ is strictly increasing and concave on $( 0,\infty ) $. As a consequence, we get some new sharp estimates to various classical inequalities concerning the gamma function and hyperbolic functions.
